# Editorial Comment: Association between Attention Deficit Hyperactivity Disorder and lower urinary tract symptoms in children: do they mean what we presume them to be?

**DOI:** 10.1590/S1677-5538.IBJU.2020.0978.1

**Published:** 2021-05-30

**Authors:** Andrew J. Combs

**Affiliations:** 1 Weill Cornell Medicine Pediatric Urodynamics in Urology New YorkNY USA Pediatric Urodynamics in Urology, Weill Cornell Medicine, New York, NY, USA

## COMMENT

In this paper the authors reported finding a statistically significant higher incidence of children with lower urinary tract symptoms (LUTS) having an association with ADHD when compared to those with LUTS without ADHD (1). Theirs was a cross sectional study in their multidisciplinary pediatric clinic who were screened for both diagnoses using standard validated questionnaire type assessment tools (DVSS for LUTS and the MTA-SNAP-IV for ADHD) with positivity for having either diagnosis resting solely on having scored at or above preset scoring criteria for each instrument. The majority of those screened were there for reasons other than behavioral or lower urinary tract issues and only a small number had been previously diagnosed with either ADHD or LUTS. They also identified the ADHD subtype (Combined type) as being most often associated with LUTS and Urgency the most common LUTS associated with ADHD. Their findings are consistent with the many previous reports in the literature on this topic as is their admonition as to the importance of screening for both and treating accordingly.

But it is also consistent with the concerns that inherently exist in studies that report on associations between different medical conditions or symptom groups (2). When a study reports an association between ADHD and LUTS it is only reasonable to ask what is the nature of that association? Is it etiologic related or is one related to the other by virtue of the confounding effect each may have. And if so, is it the same relationship for all LUTS or only certain LUTS that suggest a particular LUT Condition (LUTC) such as idiopathic detrusor overactivity, often termed OAB, where it has been postulated that the issue is a failure of central inhibition and for which treatment with methylphenidate has been advocated. Did that treatment alone resolve the issue supporting etiology, or did they still required antimuscarinic therapy to be added in order to be completely eradicated supporting it being more of a confounder? Or is it that if treatment for detrusor overactivity is delayed for a prolonged period of time, that significant injury to the bladder can occur making adjuvant therapy unavoidable? Also helpful in better understanding these relationships would be periodic ADHD symptom screening during and after therapy for their urologic issues in order to see what effect successful treatment of their LUTS/LUTC had on their initial ADHD scores, i.e. did the ADHD symptoms/parents’ perception of them improve as LUTS resolved, perhaps lowering sufficient enough to either influence the criteria for its diagnosis in mild cases or its severity grading. Time to best urologic response between those with LUTC without associated ADHD is also useful. Follow up post LUT-C treatment should also include objective reassessment post treatment to document that in fact the LUTC that was initially diagnosed was actually corrected, i.e. did reduction of LUTS in a patient with documented Dysfunctional Voiding was now voiding in a normal synergistic fashion. It is these and many similar questions that need addressing.

While the MTA-SNAP-IV is considered valuable for assessing ADHD symptom severity and plays a supporting role in the diagnostic process, it is generally considered by most clinicians in that field to not be the sole arbiter for that diagnosis and the same could be said for the DVSS questionnaire, particularly when there are no LUTS absolutely specific for any LUTC and there are many LUTS that are common in a variety of LUTCs making relying on questionnaire type instruments alone of limited value (3, 4). A good example of this has been the AUA symptom score and bothersome index. Developed decades ago it was initially intended as an objective means of establishing baseline symptoms in both men and women and subsequently monitor for changes related to both time and treatment response as well as monitor patient satisfaction. It was never intended to diagnose any specific voiding disorder or urodynamic parameter of bladder function and was clearly so stated when first coming into use. Yet over the years it has morphed from a useful monitoring tool to where now, it and its subsequent iterations are typically being used to diagnose conditions, grade severity of that condition, justify various interventions and substantiate claims of treatment efficacy, rarely without any real urodynamic evidence to support it. In general, it is not the symptoms of any particular condition or disease process that we treat per se, rather it is the condition driving those symptoms which is why diagnostic accuracy is of such importance and is underscored by the phrase “know thy enemy”.

While the literature is replete with specific objective diagnostic criteria for ADHD and its various subtypes, this is not so as regards LUTS where symptoms, sometimes bolstered by uroflow pattern appearance, are often the sole arbiters of the presumed underlying LUTC diagnosis. In that vein, included below are references that may help to better illustrate what is meant by using objective diagnostic criteria for parsing out which common LUTC is being treated even though these are by no means the only objective paradigms that can be used nor do they conform 100% with current ICCS recommended terminology.

Another problem area is that while most clinicians agree in principle that treatment should be multidisciplinary, it has been my observation that it has become increasingly prevalent that the specialty that drives the bus so to speak as to how these children are managed is the specialty to which the patient first sought care, or for whatever reason was deferred to, and that can be either Urology, Colorectal/GI or Psychiatry and can potentially have a negative effect on how quickly the child's various issues are resolved if not addressed simultaneously early on.

For those practices fortunate to be located in a center where a more centralized, multidisciplinary approach to care is feasible as in the case of these authors, there is not only adequate resources to provide all the care services needed but real potential to more scientifically investigate the true nature of these associations. In just such a setting there is also the opportunity if one were so inclined, to initiate carefully constructed investigational studies to more clearly identify whether any particular type of ADHD is the underlying etiology responsible for any specific LUT condition or are they more simply associations that act as an impediment to achieving a successful therapeutic response. If this line is pursued it will hopefully provide not only insight into the true nature of the ADHD-LUT Dysfunction association, but also lead to more refined treatment recommendations.

In the end one cannot over emphasize the importance for all clinicians who treat these children, regardless of their practice setting, to remain aware of the frequent associations between ADHD and both Bowel and Bladder disorders which if left unrecognized and addressed, can seriously undermine the optimization of patient care and that the focusing of treatment on one particular condition at the expense of the other is generally not helpful. And finally, when reporting one's results in the literature on the nature of those associations and treatment outcomes, it is best served when the reported disorders are clearly and objectively defined, that treatments be carefully applied in the order of greatest clinical need to better discern which was responsible for response and that statistically significant improvements be paired with truly meaningful clinical improvements as well. This is not to acknowledge that sometimes the exact etiologic cause of a given disorder cannot be readily proven or that often multimodal therapy is needed for optimal response, only that the further away from scientific methods of proof and the more one relies on associations drawn from inference and data that can be subjectively influenced, the more the likely the take home message will remain muddled, not clarified.
